# Changes of Quality of Minimally-Processed Pineapple (*Ananas comosus*, var. ‘Queen Victoria’) during Cold Storage: Fungi in the Leading Role

**DOI:** 10.3390/microorganisms8020185

**Published:** 2020-01-28

**Authors:** Charlène Leneveu-Jenvrin, Baptiste Quentin, Sophie Assemat, Mathilde Hoarau, Jean-Christophe Meile, Fabienne Remize

**Affiliations:** 1 QualiSud, Univ de La Réunion, CIRAD, Univ Montpellier, Montpellier SupAgro, Univ d’Avignon, 2 rue J. Wetzell, F-97490 Sainte Clotilde, France; b.quentin2605@gmail.com (B.Q.); fabienne.remize@univ-reunion.fr (F.R.); 2 CIRAD, UMR QualiSud, F-97410 Saint Pierre, La Réunion, France; sophie.assemat@cirad.fr (S.A.); meile@cirad.fr (M.H.); jean-christophe.meile@cirad.fr (J.-C.M.); 3 QualiSud, Univ Montpellier, CIRAD, Montpellier SupAgro, Univ d’Avignon, Univ de La Réunion, F-34000 Montpellier, France

**Keywords:** fruit, microbiological quality, sensory quality, fresh-cut

## Abstract

Minimally-processed pineapple stored under refrigerated conditions is highly perishable. We aimed to characterize the evolution of physicochemical, sensory and microbiological quality during cold storage. Pineapple batches were sampled from several locations in Reunion Island and then minimally processed. In the processing step, the variability of firmness and counts of yeasts and molds were observed. Moreover, correlations between the sampling season and pH and b* color component, as well as between fungal population and b* parameter were observed. During storage, the visual aspect of pineapple cuts changed to brown and shiny, whereas olfactive descriptors shifted from fruity descriptors and fresh to fermented, alcoholic and milky. The values for pH, TA and TSS did not significantly vary according to storage time. A decrease in firmness and C* color parameter was observed. Yeast and mold counts were significantly higher after 7 days of storage. The diversity in yeasts and molds was mainly dependent on the considered batches observed from PCR-DGGE profiles. Fungal species were isolated from spoiled pineapple cuts. The implication of *Penicilllium citrtrinum*, *Talaromyces amestolkiae*, *Rhodotorula mucilaginosa*, *Saccharomyces cerevisiae*, and *Meyerozyma caribbica* in the spoilage of minimally-processed pineapple cuts was further demonstrated.

## 1. Introduction

Fruit and vegetable consumption has considerably increased in the past decade. In fact, they have now become an essential part of the human diet. Pineapple (*Ananas comosus)* is appreciated for its taste and juiciness. It presents many nutritional benefits, being a good source of antioxidants, especially polyphenols, minerals, vitamin C, vitamin A, and vitamin B6 [1,2].

Minimally-processed fruits are attractive for consumers as they are perceived as healthy, fresh and convenient for use [3]. However, their shelf-life is short, typically a few days under refrigeration, because of physiological and microbiological disorders resulting from cutting the fruit.

Many attempts have been made to increase minimally-processed pineapple shelf-life [4]. Controlled storage temperature and modified atmosphere packaging are the most used technologies to preserve the quality of minimally-processed fruit [5,6,7,8,9]. Edible coatings can also prolong its shelf-life [7,10]. Physical treatments, such as UV-C, heat, ultrasound or high pressure inert gas, successfully enhanced the shelf-life of minimally-processed pineapple [11,12,13,14]. These treatments probably affected fruit enzyme activities involved in the respiration rate, browning and metabolism of phenolic compounds. The growth of microorganisms, either the natural flora [7,10], yeasts isolated from spoiled pineapple [6] or foodborne pathogens [10] was modulated by the above-mentioned treatments.

The impact of these technologies was also determined on respiration rate, firmness degradation and color changes, especially for L* and b*, which decrease reveals translucency of fruit pieces [5,7,12]. However, above all, special attention was paid to sensory properties and volatile compounds involved in the pineapple aroma [2]. From six different cultivars, 83 volatile compounds, including 15 esters and 57 alkenes, were detected [2]. Different volatile compounds were shown to be involved in the aroma [2,6,8]. During the storage of minimally-processed pineapple, an increase in concentration of ethanol, ethylacetate, acetaldehyde, and methyl esters of carboxylic acids was noticed in several studies [6,8,15]. However, some discrepancies were observed in other volatile compounds detected, possibly in relation to cultivar or storage conditions. The increase in concentration of volatile compounds observed during storage can result either from living tissue metabolism or production by yeasts [6]. The main difference in samples stored under different conditions was attributed to a sensory panel to odor, especially with the “fermented” descriptor [6]. However, Wu et al. (2012) [11] noticed that most differences were detected in appearance and flavor between control and high-pressure treated pineapple cuts.

The present study aimed to describe the initial physicochemical, sensory and microbiological qualities of minimally-processed ‘Queen Victoria’ pineapple and their changes upon storage at 4 °C. A focus was put on the diversity of yeasts and molds and the modulation of the encountered species according to the sample and the storage time.

## 2. Materials and Methods

### 2.1. Fruits Preparation

**Figure 1:**
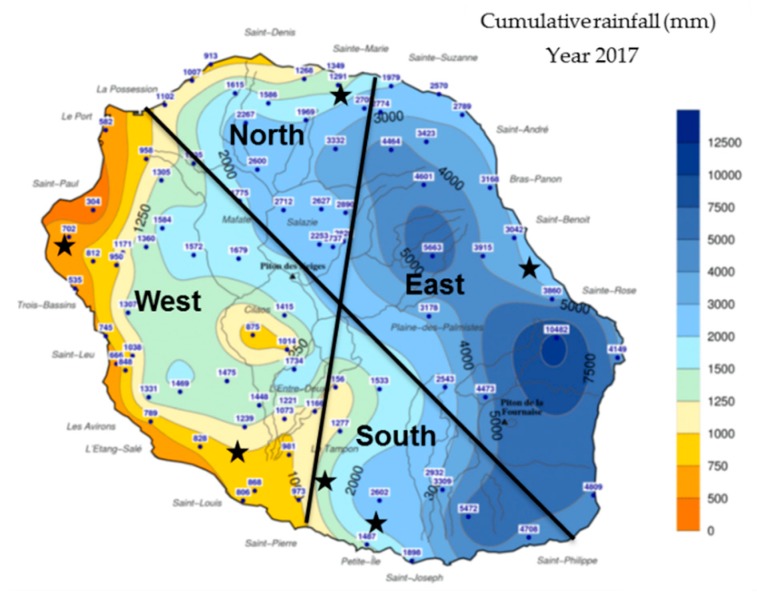
Map of Reunion Island with sampling locations (black stars). Four locations were differentiated based on annual cumulative rainfall on year 2017. Source: Meteo France.

‘Queen Victoria’ pineapples were collected from different locations in Reunion Island (Figure 1) over 2 years (2017–2019). Locations mostly differed in their annual rainfall: West location is characterized by very low rainfall (500–1000 mm), South and North locations by moderate rainfall (1250–2000 mm), and East location by very high rainfall (2000–3000 mm). Average daily solar radiation is high (1700–2100 J/cm²) in all locations. In Reunion Island, only two seasons are observed: winter from April to September, and summer from October to March.

For each sampling date and origin, at least three pineapples with similar maturity levels were picked up (Table 1).

In addition, three samples were collected from a local producer of minimally-processed fruit and vegetables (Table 1).

Within 24 h after collection, the fruits were manually peeled and cut into pieces of ca. 3 × 2 cm. Pieces from different pineapples were manually mixed and further shared into commercial freezer bags. Each bag contained ca. 100 g of fruit and was stored at 4 °C until analysis. One bag was used for each time point of analysis.

### 2.2. Fruit Quality Determination

#### 2.2.1. Microbiology

Prior to analysis, 30 g of fruits were collected from freezer bags and mixed with 30 mL of SPW (saline peptone water, Condalab, Torrejón de Ardoz, Madrid, Spain) in a stomacher for 1 min at maximal speed. For microbial analysis, serial decimal dilutions were performed in SPW. Enterobacteria were enumerated on VRBG agar (Biokar diagnostic, Solabia, Allonne, France) after incubation for 48 h at 37 °C. Psychrotrophic bacteria were enumerated on nutrient agar (Merck, Darmstadt, Germany) incubated for 3 days at 10 °C. Yeasts and molds enumeration was performed on Sabouraud glucose agar with 100 mg/L chloramphenicol (Biokar diagnostic, Solabia, Allonne, France) after incubation at 30 °C for 5 days. Yeast and molds were isolated under the same conditions.

#### 2.2.2. Physicochemical

pH value was measured by a pH meter (5231 Crison, and pH-meter Model GLP22, Crison Instruments S.A. Barcelona, Spain), and the titratable acidity (TA) was determined by titration with 0.05 M NaOH (TitroLine easy, Schott, Mainz, Germany). TA was expressed as citric acid equivalents in g/100 mL.

Pineapple total soluble solids (TSS), expressed as Brix degrees (°Brix), were determined with a hand refractometer (Atago, Tokyo, Japan) at room temperature.

Three color determinations were performed for each sample (12 mL of mixture) with a spectrophotometer CM 3500d (Minolta^®^, Carrières-sur-Seine, France). Measured color parameters were L*, a* and b*. Numerical values of a* and b* were converted into a saturation variable or chroma:

(1) $$C*=\sqrt{\left(a{*}^{2}+b{*}^{2}\right)}$$

and in a measure of chromaticity, the hue angle:

(2) h°=arctan(b*/a*)

Color difference:

(3) $$\Delta E=\sqrt{{\left(L{*}_{e}-L{*}_{c}\right)}^{2}+{\left(a{*}_{e}-a{*}_{c}\right)}^{2}+{\left(b{*}_{e}-b{*}_{c}\right)}^{2}}$$

in which L*_e_, a*_e_ and b*_e_ refer to the assay condition; and L*_c_, a*_c_ and b*_c_ to the control condition, which was calculated with the initial color as control.

Texture analysis was performed with a TA-TX2^®^ texture analyzer (Stable Micro Systems Ltd., Godalming, UK) equipped with a 5 kg load cell. A 2-mm diameter rod was used to penetrate the pineapple wedge sample at a test speed of 0.5 mm/s. The maximum penetration force was measured and taken as firmness (N). Three pieces of pineapple were randomly withdrawn from each bag to carry out repeats.

### 2.3. Spoilage Assays

Nine fungal isolates were collected from pineapple cuts and identified (Table 2). Their ability to spoil commercial pineapple juice (pasteurized, LawLam^®^) was assayed by the inoculation of juice and observation of changes during storage. Ten milliliters of juice were inoculated with ca. 4.6 log CFU/mL of each fungal isolate and stored for 7 days at 4 °C. Fungal enumeration and observation of color modification, gas production and off-odors production were performed to detect spoilage activity. This detection was performed in triplicate.

To confirm the ability of each isolate to spoil minimally-processed pineapple, selected isolates were separately prepared and used for inoculation. Pineapple cuts were dipped in an aqueous solution containing ca. 4.6 log CFU/mL of fungal spoilage cocktail (1:2 *w*/*v*) for 5 min at 150 rpm. Afterwards, they were drained and packaged (100 ± 2 g) in freezing bags and stored for 7 days at 4 °C. Fruit quality was then determined.

### 2.4. Sensory Quality Characteristics

Sensory quality of ripe pineapple cuts was carried out by a panel of trained judges. Pineapple cuts were placed at room temperature 1 hour before sensory analysis. Three pieces per sample were randomly served and arranged on the plate for evaluation. Each sample was coded differently, with a three-digit code.

Descriptive profiles were determined for each sample. Preliminary sessions enabled to generate a descriptive pineapple vocabulary. An 11-point scale between 0 and 10 was used to rate the intensity of the different sensory criteria. The ISO 11035 (Sensory analysis—Identification and selection of descriptors for establishing a sensory profile by a multidimensional approach) method was employed.

Two distinct tests were used to determine if there was a detectable difference between two products A and B: the two/five test and the triangle test. In the two/five test, samples from the same batch stored for 0, 3, 7 or 14 days were compared. The triangle test was used to compare the control sample and sample treated with the selected fungal cocktail, both after 7 days of storage according to the sensory method described by ISO 4120-2004 (Sensory analysis–Methodology–Triangle test).

### 2.5. Molecular Biology Methods

#### 2.5.1. DNA Extraction

DNA was extracted for each pineapple sample from the SPW suspension prepared for microbial counting. To achieve extraction, 2 mL of the suspension (stored at −80 °C) were collected in two microtubes and centrifuged at 14,000× *g* during 2 min. DNA extractions were carried out on the assembled pellets according to procedure described by MP Biomedicals^®^ (llkirch, France) with the FastDNA kit and the FastPrep-24 Instrument using Lysing Matrix A and Lysis buffer CL-Y.

#### 2.5.2. PCR-Denaturing Gradient Gel Electrophoresis (DGGE)

The following DNA primers were used to amplify a region of the fungal ITS: GC-ITS1F (CGCCCGCCGCGCGCGGCGGGCGGGGCGGGGGCACGGGGGGCTTGGTCATTTAGAGGAAGTAA) and ITS4 (TCCTCCGCTTATTGATATGC) primers [16]. A 40-pb GC-clamp was added to the forward primer in order to ensure that the fragment of DNA remains partially double stranded and that the region screened is in the lowest melting domain [17].

PCR amplification reaction was performed in a final volume of 50 μL containing 0.6 μM of each primer, all the deoxyribonucleotide triphosphate (dNTPs) at 200 μM, 2 mM of MgCl_2_, 10 μL of 5X *Taq* reaction buffer (Promega, Charbonnières-les-Bains, France), 1.25 U of *Taq* DNA polymerase (Promega), and 1 μL of extracted DNA. PCR amplification reactions were carried out as follows: an initial denaturation at 95 °C for 2 min, 40 cycles at 95 °C for 15 s, 57 °C for 30 s and 72 °C for 45 s, and final extension at 72 °C for 5 min. The PCR reactions were performed in a Thermocycler (Veriti, Applied Biosystems, Thermo Fisher Scientific, Loughborough, UK). Aliquots (5 μL) of PCR products were checked by electrophoresis migration in 2% (*w*/*v*) agarose gel with 1× TAE buffer (40 mM Tris-HCl pH 7.4, 20 mM sodium acetate, 1.0 mM Na_2_-EDTA). After running at 100 V for 45 min, the gels were stained with ethidium bromide solution (50 μg/mL in TAE 1×) and quantified using a standard (DNA mass ladder 100 bp, Promega).

The PCR products were separated by DGGE using a Cleaver Scientific system (Cleaver Scientific, Rugby, Warwickshire, UK). Briefly, 30 µL of PCR amplicons were loaded onto 8% (*w*/*v*) polyacrylamide gels (acrylamide: *N*,*N*-methylene bisacrylamide, 37.5:1, Promega) in 1× TAE buffer (40 mM Tris-HCl pH 7.4, 20 mM sodium acetate, 1.0 mM Na_2_-EDTA). Electrophoresis was performed at 60 °C using a denaturing gradient ranging from 40% to 70% (100% corresponded to 7 M urea and 40% *v*/*v* formamide, Promega). The gels were run at 20 V for 10 min and then at 80 V for 16 h. After electrophoresis, the gels were stained for 1 h with ethidium bromide solution (50 μg/mL in 1× TAE), rinsed for 1 h in distilled water, and then photographed on a UV transilluminator (Bio-Rad, Marnes-la-Coquette, France). Bands of interest were cut, and the DNA was extracted. Electrophoretic profiles were analyzed with Phoretix 1D Pro software (Totallab, Newcastle-Upon-Tyne, UK), which considers band presence and relative intensity on the line. Nearest Neighbor algorithm and Pearson coefficient correlation were used to build the dendrogram.

Detected bands were cut from the DGGE gel with a sterile scalpel as described previously [18]. Briefly, the DNA of each band was then eluted in 100 µL of TE buffer (10 mM TrisHCl; 1 mM EDTA; pH 7.4, Sigma-Aldrich, Lyon, France) at 4 °C overnight. DNA was precipitated by adding 1/10 volume of sodium acetate (3 M, pH 5), 1µL of glycogen (Molecular Grade, Roche Diagnostics, Meylan, France), and 300 µL of isopropanol and centrifuged at 15,000× *g* for 30 min at 4 °C. The supernatant was discarded, DNA pellets were washed with 500 µL 70% ethanol and after 5 min of centrifugation, the DNA pellets were air dried for 1 h. Finally, the DNA was re-suspended in 20 µL of ultrapure water and stored at −20 °C.

#### 2.5.3. Identification

DNA primers ITS1F (CTTGGTCATTTAGAGGAAGTAA) and ITS4 were used to amplify a region of the fungal ITS [16]. PCR amplification reaction was performed in a final volume of 50 μL containing 0.3 μM of each primer, all the deoxyribonucleotide triphosphate (dNTPs) at 200 μM, 1.5mM of MgCl_2_, 10 μL of 5× *Taq* reaction buffer (Promega), 1.5 U of *Taq* DNA polymerase (Promega), and 1 μL of extracted DNA. PCR amplification reactions were carried out as follows: an initial denaturation at 95 °C for 2 min, 40 cycles at 95 °C for 15 s, 53 °C for 30 s and 72 °C for 45 s, and final extension at 72 °C for 5 min. The PCR reactions were performed in a Thermocycler (Veriti, Applied Biosystems, UK). Aliquots (5 μL) of PCR products were analyzed by electrophoresis in 2% (*w*/*v*) agarose gel with 1× TAE buffer (40mM Tris-HCl pH 7.4, 20mM sodium acetate, 1.0mM Na_2_-EDTA).

After running at 100 V for 45 min, the gels were stained with ethidium bromide solution (50 μg/ mL in TAE 1×) and quantified using a molecular weight marker (100 bp DNA ladder, Promega).

The PCR products were sent to Macrogen (Amsterdam, The Netherlands) for sequencing. For each, purification was applied, and sequencing was carried out on PCR products using ITS1F or ITS4 primers. The sequences obtained were aligned with BioEdit software (Sequence Alignment Editor version 7.1.3.0, Freeware Copyright 1991–2007 Tom Hall) followed by a BLAST similarity search.

### 2.6. Statistical Analysis

The XLSTAT software (Addinsoft, Paris, France) was used for statistical analyses.

One-way variance analysis (ANOVA) was performed with a *p*-value of 0.001. The Ryan-Einot-Gabriel-Welsh F (REGWQ) test was used for pair-wise comparisons.

A correlation test for quantitative variables was performed with Kendall’s tau coefficient with a *p*-value of 0.05. The correlation between one quantitative and one qualitative variable was investigated with the biserial correlation method, using the Monte-Carlo simulation.

Word cloud was used to visualize the frequency of descriptors chosen by the panel for samples with the same duration of cold storage: Word font size is proportional to frequency. *K*-means classification was used to gather samples into classes according to their description with quantitative variables. Two-dimension principal component analysis (PCA) plot was used to represent variables (olfactive descriptors), observations (minimally-processed pineapple stored for 0, 3, 7 or 14 days) and their relatedness and distance.

## 3. Results

### 3.1. Freshly Prepared Minimally-Processed Pineapple Characteristics

A sensory analysis was performed to assign descriptors to freshly minimally-processed samples. The major color descriptor was “yellow”, and olfactive descriptors were, in descending order of frequency of occurrence, “pineapple”, “fresh”, “fruity”, “pomegranate”, “red fruit”, “citrus”, and “sugared”. These olfactive descriptors are primarily related to fruity characteristics. This is consistent with previous studies showing the role of methyl esters and lactones in the typical fruity flavor of pineapple [19,20].

The 25 pineapple batches were independently minimally-processed. For each, pH, TA, TSS, firmness, L*, a*, and b*, color parameters were determined on the day of processing. Psychrotrophic bacteria, enterobacteria, as well as yeasts and molds were counted. Table 3 shows mean values and data dispersion between batches.

The pH values varied between 3.09 and 4.20, and 64% of them ranged between 3.3 and 3.8. Similarly, for TA, 68% of values were in the range 0.73 and 0.98 g/100 mL. The values for pH, TA, and TSS were in accordance with previously published data of carbohydrate content of pineapple [1,2,7,10,21,22].

Firmness range was large, from 2.0 to 6.7 N. Among all parameters, firmness exhibited the highest variation coefficient, of 32%. This range is in accordance with literature data.

For the L* parameter, except for one extremely high value, all batches exhibited values below 41.6. On the opposite, a* values were spread on the whole range, whereas 85% of b* values were above 45. As a consequence, C* and h° exhibited a Normal distribution, with *p*-values, calculated with the Anderson-Darling test, of 0.53 and 0.23, respectively.

Counts of psychrotrophic bacteria were for most batches below the detection limit. However, five batches exhibited counts above 4 log CFU/g. For yeasts and molds, 72% of batches exhibited counts above 4 log CFU/g, and 16% above 5 log CFU/g. Eventually, for enterobacteria, 40% of batches exhibited counts above 4 log CFU/g, and 8% above 5 log CFU/g. The highest counts were thus observed for yeasts and molds.

Correlations between independent quantitative variables (pH, TA, TSS, microbial counts, L*, a*, b*, firmness) were searched with the non-parametric Kendall test. A positive correlation between L* and psychrotrophic bacteria enumeration was detected with a *p*-value of 0.028 and a Kendall’s tau coefficient of 0.345. Two negative correlations, between pH and L* and between yeast and mold counts and b*, were detected with *p*-values of 0.021 and 0.044 and coefficients of -0.336 and -0.291, respectively. Surprisingly, no correlation was pointed out between TA and TSS, which evolve in an opposite way during fruit ripening.

**Figure 2:**
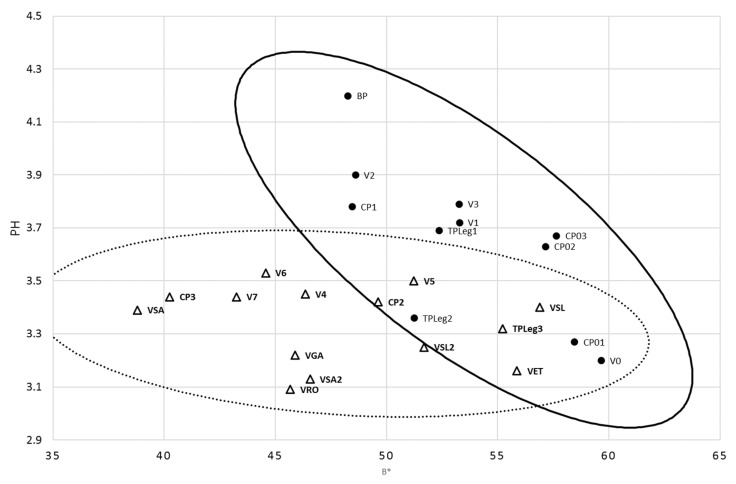
Relationship between season and the parameters pH and b* from fresh minimally-processed pineapple. Black circle: summer; white triangle: winter. Confidence ellipses correspond to the seasons.

Eventually, correlations were searched between qualitative (season and location of sampling) and quantitative variables. Biserial correlation tool showed a correlation between season and pH, with a *p*-value of 0.0002 and a coefficient of 0.60, and a correlation between season and b*, with a *p*-value of 0.015 and a coefficient of 0.49. This grouping is visualized from pineapple batches plotted on a graph with *x*-axis being b* and y-axis being pH (Figure 2).

Harvesting season has considerable impacts on the post-harvest quality of pineapple, affecting internal browning and storage life [19]. The correlation between season and pH is not surprising but could have been expected also with TA and TSS. Pineapple flesh color, especially b* and C*, TSS, TA, and pH were influenced by the season in Thailand (three seasons: summer, rainy and winter) for the Smooth Cayenne cultivar [23]. The importance of pre-harvest factors on post-harvest quality was underlined by Chen et al. (2009) [24]. Whereas a model has been proposed to predict TSS of ‘Queen Victoria’ pineapple flesh from agroclimatic conditions of Reunion Island [21], no correlation has been previously pointed out between pH, b* color parameter and season, for this cultivar or crop location.

The relationship between pH and L* could be explicated by different pineapple flesh compositions, which are reflected on these two parameters. Pineapple flesh color depends on its composition in carotenoids and flavonoids, and the content in those compounds was showed to depend on agroclimatic conditions [25]. Moreover, the color of flavonoids depends on the pH. By contrast, explaining the correlations between a color parameter and a microbial count would require extensive metabolomic analyses to find out which compounds would be implied.

### 3.2. Minimally-Processed Pineapple Changes over Refrigerated Storage

**Figure 3:**
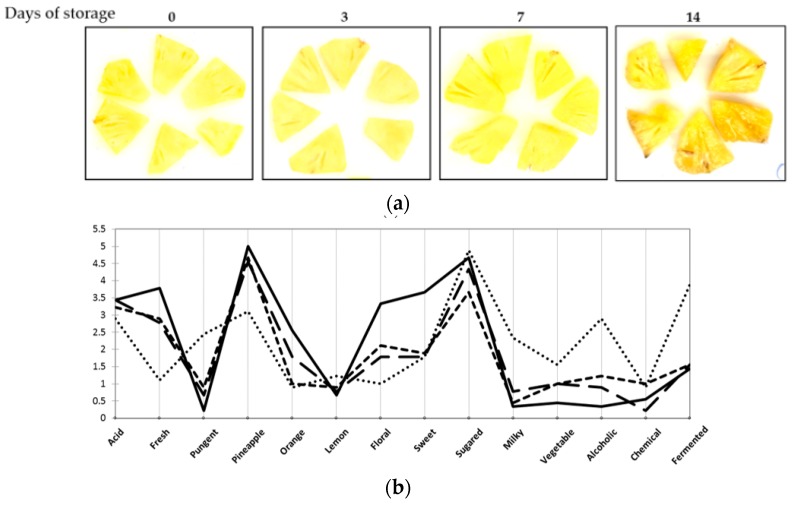

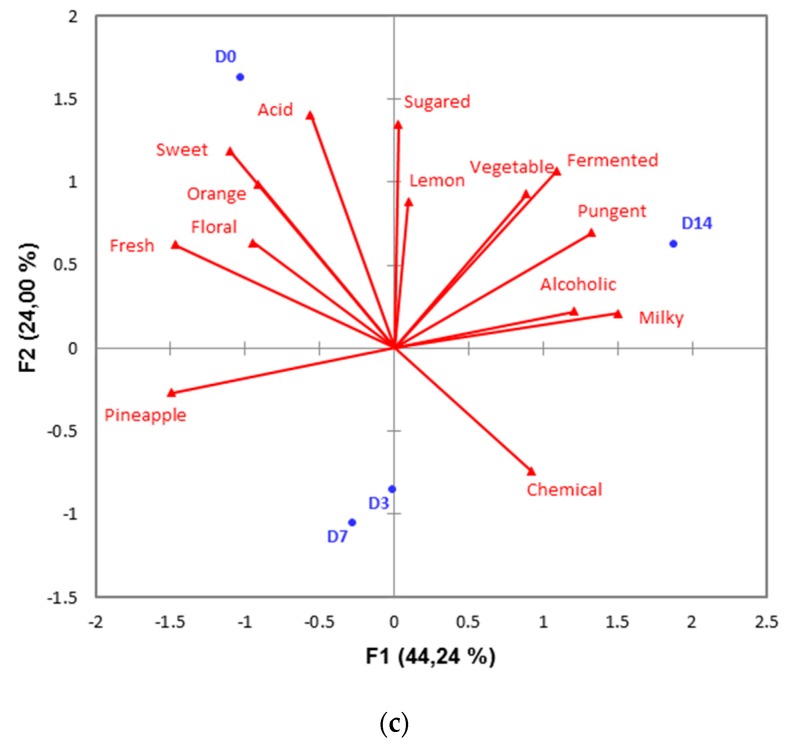
(**a**) Visual aspect of pineapple cuts during storage. From left to right: 0, 3, 7 and 14 days of storage at 4 °C; (**b**) Olfactive descriptive profiles of minimally-processed pineapple stored at 4 °C for 0 days (plain line), 3 days (large dashes), 7 days (short dashes) or 14 days (dotted line); (**c**) PCA analysis of olfactive profiles. Sum of F1 and F2 represents 68.24% of data. D0, D3, D7, D14: samples stored at 4 °C for 0, 3, 7, and 14 days respectively.

Sensory descriptive profiles (olfactory and aspect) were established on minimally-processed pineapple during refrigerated storage, after 3, 7, 10, and 14 days, and compared to freshly processed samples (Figure 3).

The visual aspect of minimally-processed pineapple clearly turned slightly brown and shiny after 14 days at 4 °C (Figure 3a). K-means classification positioned samples into four classes (Figure 3b). Day 0 samples (fresh pineapple) were in class 1. Olfactive descriptors of samples from day 14 were “fermented”, “pungent”, “alcoholic”, “vegetable”, and “milky”. These descriptors indicate a negative evolution of sensory quality of the product over time. They can be related to a previously described increase in volatile organic compounds such as ethyl acetate, acetic acid, ethanol or palmitic acid during storage of pineapple cuts [19,26]. Significant differences were observed between freshly prepared and (class 4) 14-day stored samples. This accounts for “fresh”, “pineapple”, “pungent”, and “chemical” descriptors. Samples from day 3 and 7, respectively in classes 2 and 3, were mainly characterized by “acid”, “fermented” and “chemical”. PCA analysis showed clearly the differences depending on the storage time of samples determined from olfactive descriptors (Figure 3c).

Depending on the batch, quicker spoilage could be observed (data not shown) and thus analyses were stopped when a spoilage was observed. For instance, batches TP1, TP2 and TP3, which came from the same place, were not acceptable after 7 days of storage. Batches BP, CP01, CP02, CP03, CP1, V0, V1, V2, and V3 were not acceptable after 14 days of storage. The common feature of the latter batches is that they were all sampled during the summer season. All winter batches, except TP3, were considered as acceptable after 14 days of storage.

**Figure 4:**
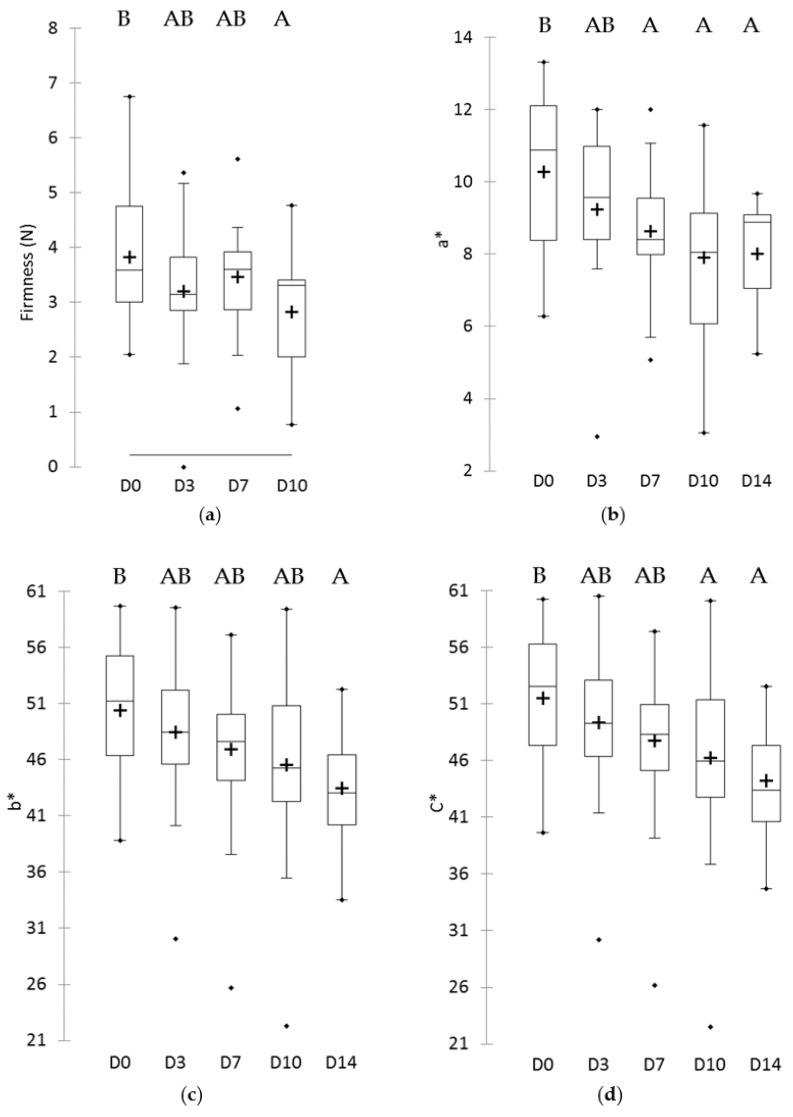
Values for (**a**) firmness, (**b**) a*, (**c**) b* and, (**d**) C* color parameters of 25 minimally-processed pineapple batches during storage at 4 °C. Time of storage varies from 0 (D0), 3 (D3), 7 (D7), 10 (D10), and 14 (D14) days. (+) indicates the mean value, and black dots the outliers. For each parameter, different uppercase letters indicate significant differences between storage times.

Changes in physicochemical parameters were determined over the shelf-life of minimally-processed pineapple (Figure 4 and Figure S1). The values for pH, TA and TSS did not significantly vary according to storage time. On the opposite side, storage time influenced firmness and color parameters. A decrease of 26% of firmness was observed after 14 days, when compared to the determination performed the day of processing. The L* value was not modified according to storage time, but a* and b* decreased. The calculated dependent parameter C* decreased over storage time, but h° and ΔE did not vary significantly.

Some physicochemical parameter changes of minimally-processed pineapple during cold storage have been described [5,7,8,10,12,27,28,29,30]. In most of these studies, pH appeared stable or varying by less than 0.2 units over storage time. For TSS, conflicting results are reported, decreasing [28], stable [27] or increasing [10] during storage. A gradual decrease in firmness was observed previously [5,10,30]. Changes of color, especially browning, have been described during the shelf-life of minimally-processed pineapple. A a* value increase was observed during the first 8 days of storage in several studies [10,11,29], whereas a sharp L* and b* values decrease was observed in other studies [7,10,12,30]. The most reproducible changes are thus pH and TA stability, firmness decrease and b* decrease, indicating a loss of the yellow color because of either browning or translucency.

Counts of psychrotrophic bacteria did not increase during storage time, with mean values of 3.6 to 3.9 log CFU/g at each sampling time (Table 4). This observation hides great differences between samples, most of samples exhibiting counts below the detection limit and some of them showing counts up to 6.9 log CFU/g after 10 days of storage. Consequently, a moderate increase of ca. 1.5 log CFU/g would not have been detected as significant in our experimental conditions. It was showed that psychrotrophic bacteria counts increased by less than 2 log CFU/g during 12 to 14 days of storage of pineapple cuts [7,11]. Counts of enterobacteria remained stable at 3.9 log CFU/g during the first 7 days and increased thereafter to reach 4.5 log CFU/g after 14 days of storage (Table 4). The maximal observed value of enterobacteria counts gradually increased during storage. Lastly, yeast and mold counts gradually increased during storage time, from initially 4.4 log CFU/g to 5.1 log CFU/g after 7 days and 6.0 log CFU/g after 14 days. Both minimal and maximal yeast and molds counts changed during storage time to reach respectively 4.0 log CFU/g and 7.6 log CFU/g after 10 days, and 5.0 log CFU/g and 7.9 log CFU/g after 14 days (Table 4). Significant differences depending on storage duration were observed for yeast and mold populations.

Fungal population was compared after 3 days of storage according to the visual spoilage observed. Batches TP1, TP2 and TP3, which appeared spoiled after 7 days of storage, exhibited a fungal population after 3 days of 5.6 log CFU/g. Batches spoiled after 14 days of storage (BP, CP01, CP02, CP03, CP1, V0, V1, V2, and V3) showed fungal counts of 4.8 log CFU/g after 3 days. The last group of batches, not spoiled after 14 days of storage, exhibited a mean fungal population after 3 days of 4.8 log CFU/g, and thus were identical to the latter group. Yeast and mold counts cannot be strictly correlated to shelf-life.

Mesophilic and psychrotrophic bacteria are possibly involved in minimally-processed pineapple spoilage, whereas enterobacteria enumerations were used as hygienic indicators of processing. In our study, the abundance of the two bacterial groups was monitored and did not increase significantly. The growth of mesophilic and psychrotrophic bacteria in minimally-processed pineapple has been reported, but consistently to a lesser extent than yeasts and molds [7,10,27,28]. A large and rapid increase of yeast and mold counts has been previously observed during cold storage of fresh-cut pineapple [7,10,27,29], confirming our observation. Yeasts and molds are reported as the main contaminant of fruit salads and fruit juices [31,32]. Yeasts and molds are favored by the high sugar content and the pH values, comprising between 3.09 and 4.20 for all batches, of minimally-processed pineapple. They can be responsible for spoilage by producing gas, ethanol and volatile compounds with off-odors.

Moreover, we showed that fungal counts cannot be solely used as a spoilage indicator, as they are not strictly correlated to shelf-life. For that reason, yeast and mold diversity and the relationship to spoilage was focused on.

### 3.3. Diversity of Yeasts and Molds and Modulation during Storage

**Figure 5:**
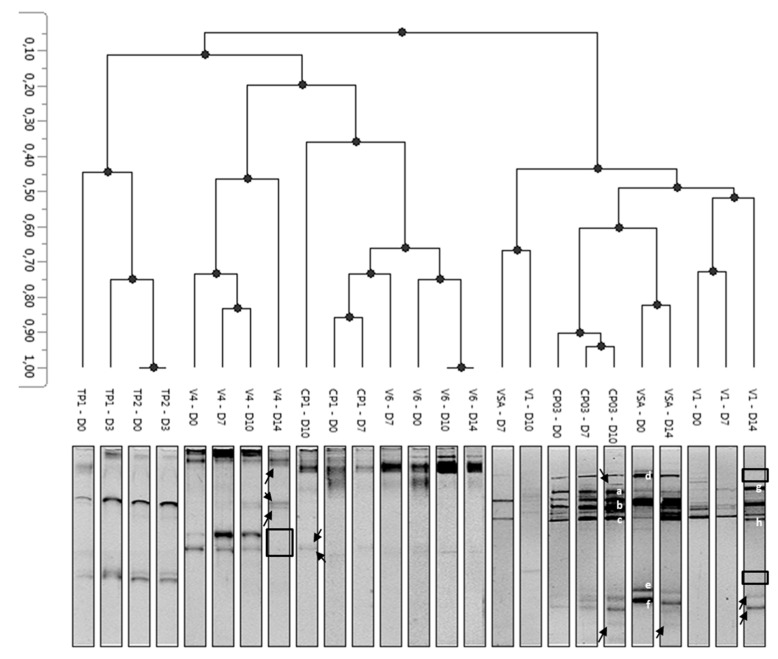
UPGMA dendrogram of PCR-DGGE profiles obtained for right batches of minimally-processed pineapple. D0, D3, D7, D10, and D14 correspond respectively to 0, 3, 7, 10, and 14 days of storage. Letters “a” to “h” label DNA bands which were sequenced. Arrows show bands appearing during storage and open boxes bands which disappear during storage.

The profile of yeast and mold communities during refrigerated storage of minimally processed pineapple was determined to see if differences between samples could be observed. To that aim, PCR-DGGE analysis was applied to eight batches (Figure 5): CP1 sampled in East area in summer, VSA sampled in East area in winter, CP03 sampled in the North in summer, V1 sampled in the South area in summer, V4 and V6 sampled in the South area in winter, and TP1 and TP2 sampled from a local producer in summer. V1, V4 and V6 originated from the same producer.

We observed that samples were primarily gathered according to the pineapple batch, rather than according to storage time. Three groups were differentiated.

The first group contained TP1 and TP2 batches that presented only four bands per lane. For these two batches, a rapid spoilage occurred and both enterobacteria and yeast and mold counts were above 5.5 log CFU/g after 3 days of storage.

The second group gathered batches V4, CP1 and V6. For those batches, most of the bands were observed at the top of the gel. An evolution of the main fungal communities was observed during storage, with the appearance (black arrow) or disappearance (empty square) of DNA bands at the latter storage times (days 10 and 14).

Lastly, the third group gathered samples CP03, VSA and V1. Their profiles were similar for five bands, which were labelled “a”, “b”, “c”, “d”, and “g”. The “d” band disappeared from V1 profile after 14 days (Figure 5). DNA retrieved from these bands and sequenced resulted in the identification of (a) *Resinicium saccharicola* (mold), (b) *Cladosporium sphaerospermum* (mold), (c) *Cladosporium cladosporioides* (mold), (d) *Disporotrichum dimorphosporum* (mold), and (g) *Rhizopycnis vagum* (mold) respectively (Table 5). The band labelled “h” was only present in V1 sample after 14 days of storage and identified as belonging to the *Rhodotorula glutinis* species (pink yeast) (Figure 5 and Table 5). In VSA and V1 groups, two specific bands were labelled “e” and “f”, and were identified as *Galactomyces candidum* (mold) and *Clavispora lusitaniae* (yeast), respectively.

Except for TP1 and TP2 coming from the same producer and exhibiting a short shelf-life, PCR-DGGE grouping was not related to sampling season, neither to the location or producer, as it can be specifically seen from V1, V4 and V6.

*R. saccharicola* was isolated from sugar cane [33]. *Cladosporium* spp. is widely present on plant material and can cause post-harvest spoilage of fruit [34,35,36,37,38], even at a low temperature. *D. dimorphosporum* is industrially used as a producer of plant cell wall lytic enzymes [39,40]. *R. vagum*, infecting roots and tubers, is known to contribute to vine decline and root necrosis [41,42,43]. *G. candidus,* teleomorph of *Geotrichum candidum*, is mainly derived from cheese [44], but also from fruit tree phyllosphere [45]. It was identified from necrotic lesions of pineapple [46]. *C. lusitaniae* was identified in apple juice and on rotten fruit [47,48,49]. *Rhodotorula* spp., corresponding to the “h” band that appeared in the V1 sample after 14 days of storage, contaminates at high levels fruit salads and juices made from cantaloupe, citrus, honeydew, strawberry, coconut water, grape, and apple [31,47,48,50]. All detected fungal species of this study have already been associated to different fruit or plant materials.

### 3.4. Identification of Fungal Isolates Involved in Spoilage of Minimally-Processed Pineapple

Nine fungal isolates were obtained from six minimally-processed pineapple batches at different storage times. Identification is proposed in Table 2. “R” and “S” colony phenotypes were similar and identified as *Penicillium citrinum* mold species. *Talaromyces amestolkiae* was another isolated mold. *Rhodotorula mucilaginosa, Saccharomyces cerevisiae*, and *Meyerozyma caribbica* corresponded to yeast species isolated. *Rhodotorula* was the only genus also identified from PCR-DGGE profiles.

*P. citrinum* has been identified from jujube (*Ziziphus mauritiana*), acid food products from citrus, coco milk, coffee and cocoa beans, in which its strong polygalacturonase activity was detected [38,51,52,53,54]. This fungus can produce the mycotoxin citrinin. *Talaromyces* spp. can occasionally be isolated from low-pH juices [55,56]. On the opposite, *R. mucilaginosa* and *S. cerevisiae* are commonly involved in the spoilage of fruit products [48,51,57,58]. *M. caribbica* is mostly known as an endophyte yeast, but has been isolated from spoiled minimally-processed pineapple [26,59].

Five isolates were selected from DNA identification and colony morphology: *P. citrinum* (R), *T. amestolkiae* (20), *R. mucilaginosa* (A), *S. cerevisiae* (C), and *M. caribbica* (F). Their involvement in spoilage of minimally-processed pineapple during refrigerated storage was investigated. Pineapple cuts, dipped in the fungal cocktail composed of the five isolates, were analyzed and compared to control cuts (not dipped) after 7 days of storage, in triplicate.

**Figure 6:**
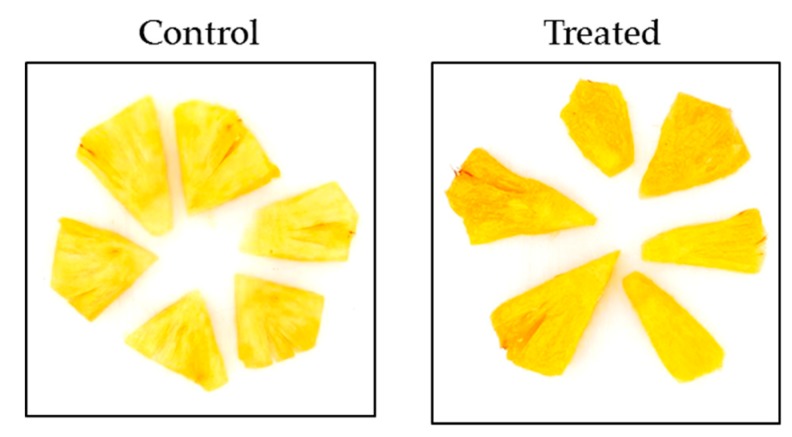
Visual aspect of pineapple cuts after 7 days of storage at 4 °C (left control, right treated).

Visually, treated cuts appeared darker (Figure 6). Sensory triangle tests confirmed the difference, with a 99.99% confidence. The panel proposed descriptors of the treated samples: These descriptors were mainly negative adjectives, “fermented” and “putrid” being the most frequent. The descriptors “milky”, “Roquefort cheese”, then “sugared” and “toffee” were less frequently cited.

The comparison of physicochemical parameters pointed to large differences between the control and the treated samples after 7 days of storage (Table 6). As expected, pH and TSS did not significantly change. On the opposite, TA decreased during cold-storage of control and increased for the treated samples after 7 days of storage. Color analysis showed that a* and Hue angle were significantly higher for the treated samples than for the control conditions. For L*, b*, C*, and ΔE, a bilateral Dunnet’s test pointed out differences between the treated samples and the control ones after 7 days of storage.

As expected, the fungal population increased for the control condition during storage, and the increase was much more marked for the treated sample (Table 6). Colony phenotypes of the five isolates were observed on enumeration media for the 7-day stored treated samples.

## 4. Conclusions

Physicochemical characteristics of minimally-processed pineapple fruit are modulated by the harvesting season. Hence, microbial populations are probably influenced by composition, together with environmental conditions. We observed that fungal diversity varied greatly according to the harvested pineapple batch.

Olfactive descriptors of minimally-processed ‘Queen Victoria’ pineapple were mostly “fresh”, “sugared” and “pineapple”. After a 14-day cold-storage, the frequency of descriptors “fermented” and “alcoholic” increased, as opposed to “pineapple” and “fresh”. At the same time, the fungal population increased to a large extent, and PCR-DGGE profiles were slightly modified. Identification of yeasts and molds from PCR-DGGE profiles and from isolates pointed to species already described as associated to carposphere or fruit foods or beverages, and for some involved in spoilage. The fungal diversity, not only population level, probably played a crucial role in triggering spoilage.

A cocktail of two molds and three yeasts was inoculated on pineapple cuts and resulted in sensory quality defects and color changes. Future research is needed to better link the presence, growth and activity of fungal species to spoilage, and hence to extend the shelf-life of minimally-processed fruit.

## Figures and Tables

**Table 1:** Pineapple sample names, sampling location and month of sampling (season).

Samples	Location	Date
**V0**	East	October 2017 (summer)
**CP1**	East	March 2018 (summer)
**VSA**	East	May 2018 (winter)
**CP3**	East	June 2018 (winter)
**VSA2**	East	June 2018 (winter)
**VET**	East	July 2018 (winter)
**VRO**	East	July 2018 (winter)
**VGA**	East	July 2018 (winter)
**CP01**	West	October 2017 (summer)
**CP02**	West	October 2017 (summer)
**V3**	West	March 2018 (summer)
**CP2**	West	April 2018 (winter)
**V5**	West	April 2018 (winter)
**VSL**	West	May 2018 (winter)
**V7**	West	May 2018 (winter)
**VSL2**	West	June 2018 (winter)
**CP03**	North	December 2017 (summer)
**BP**	South	March 2018 (summer)
**V1**	South	March 2018 (summer)
**V2**	South	March 2018 (summer)
**V4**	South	April 2018 (winter)
**V6**	South	May 2018 (winter)
**TP1**	Any	January 2019 (summer)
**TP2**	Any	February 2019 (summer)
**TP3**	Any	May 2019 (winter)

**Table 2:** Identification of fungal isolates.

Name	Identification	Sampling Source	Fragment (pb)	Identity (%)	*E*-Value	Accession Number
**R**	*Penicillium citrinum*	VSL-D14	339	98	6.0 × 10^−134^	MN046972.1
**S**	*Penicillium citrinum*	VSA-D14	426	100	0.0	MN653150.1
**A**	*Rhodotorula mucilaginosa*	CP1-D7	234	100	2.0 × 10^−117^	MN535021.1
**C**	*Saccharomyces cerevisiae*	BP-D3	507	99	1.0 × 10^−161^	MN244399.1
**D**	*Meyerozyma caribbica*	V2-D7	330	100	1.0 × 10^−170^	MN658754.1
**F**	*Meyerozyma caribbica*	VSL-D3	268	99	6.0 × 10^−133^	MN658754.1
**H**	*Meyerozyma caribbica*	CP1-D3	320	100	4.0 × 10^−165^	MN416286.1
**I**	*Meyerozyma caribbica*	BP-D3	274	100	1.0 × 10^−139^	MN658754.1
**20**	*Talaromyces amestolkiae*	TP1-D3	159	95.6	4.0 × 10^−64^	MN549518.1

**Table 3:** Values and dispersion of physicochemical parameters and microbiological counts of minimally-processed pineapple batches.

Parameter	Mean	±	Standard Deviation	Minimum Value	Maximum Value
pH	3.44	±	0.27	3.09	4.20
TA (g/100 mL)	0.87	±	0.20	0.68	1.33
TSS (°Brix)	14.6	±	1.5	11.1	17.8
L*	36.7	±	6.6	32.1	65.3
a*	10.3	±	2.1	6.3	13.3
b*	50.4	±	5.7	38.8	59.7
Chroma	51.5	±	5.6	39.6	60.2
Hue angle	11.6	±	2.5	6.3	15.3
Firmness (N)	3.8	±	1.2	2.0	6.7
Psychrotrophic bacteria (log CFU/g)	3.7	±	0.6	3.3 ^1^	5.4
Enterobacteria (log CFU/g)	3.8	±	0.6	3.0 ^2^	5.3
Yeasts & Molds (log CFU/g)	4.4	±	0.7	3.0	5.8

^1^ and ^2^: the indicated values correspond to the detection level. For psychotropic bacteria, 14 batches showed counts below this level. For enterobacteria, four batches showed counts below this level.

**Table 4:** Changes in microbial population during storage time at 4 °C.

Days of storage	0	3	7	10	14
Number of samples	25	25	22	22	13
Microbial counts in log CF/g: mean (minimum value; maximum value]					
Psychrotrophic bacteria	3.7 [3.3; 5.4]	3.7 [3.3; 5.1]	3.6 [3.3; 6.3]	3.9 [3.3; 6.9]	3.7 [3.3; 5.3]
Enterobacteria	3.9 [3.0; 5.3]	3.9 [3.0; 6.1]	3.9 [3.0; 6.1]	4.2 [3.0; 7.0]	4.5 [3.0; 7.8]
Yeasts and molds	4.4 [3.0; 5.6] A	4.9 [3.5; 6.0] AB	5.1 [3.3;7.4] B	5.5 [4.0; 7.6] BC	6.0 [5.0; 7.9] C

**Table 5:** Identification of fungal species from DNA extracted from DGGE gels.

Band	Origin	Identification	%Identity	*E*-Value
a	CP03–D10	*Resinicium saccharicola*	98	0
b	CP03–D10	*Cladosporium sphaerospermum*	96.1	0
c	CP03–D10	*Cladosporium cladosporioides*	93.9	4.0 × 10^−143^
d	VSA–D0	*Disporotrichum dimorphosporum*	85	6.0 × 10^−8^
e	VSA–D0	*Galactomyces candidu* *s*	98.8	4.0 × 10^−163^
f	VSA–D0	*Clavispora lusitaniae*	96.4	3.0 × 10^−137^
g	V1–D14	*Rhizopycnis vagum*	95.7	4.0 × 10^−146^
h	V1–D14	*Rhodotorula glutinis*	99	2.0 × 10^−86^

**Table 6:** Physicochemical parameters and population of yeast and molds for control and treated samples during storage at 4 °C. D0 correspond to freshly prepared pineapple and D7 to samples after 7 days of storage. Different uppercase letters in the same line indicate significant differences. Lower case letters indicate differences according to bilateral Dunnet’s test. *: not applicable.

Parameter	Control—D0	Control—D7	Treated—D7
pH	3.6 ± 0.1 A	3.6 ± 0.1 A	3.5 ± 0.1 A
TA (g/100 mL)	6.1 ± 0.1 B	5.4 ± 0.2 A	6.6 ± 0.2 C
TSS (°Brix)	17.1 ± 0.4 A	17.5 ± 0.5 A	16.0 ± 0.2 A
L*	39.5 ± 0.4	39.2 ± 0.8 b	34.6 ± 0.4 b
a*	10.1 ± 0.3 A	10.7 ± 0.4 A	14.2 ± 0.6 B
b*	64.8 ± 0.7	64.0 ± 1.4 b	58.0 ± 0.5 a
Chroma	65.6 ± 0.7	64.9 ± 1.3 b	59.8 ± 0.5 a
Hue angle	8.8 ± 0.3 A	9.5 ± 0.5 A	13.8 ± 0.6 B
Color difference	- *	1.2 ± 1.1 b	9.4 ± 1.4 a
Yeasts & Molds (log CFU/g)	4.8 ± 0.2 A	6.1 ± 0.1 AB	10.0 ± 0.1 B

## Supplementary Materials

The following are available online at https://www.mdpi.com/2076-2607/8/2/185/s1.
