# Supplementary material for: Changes of Quality of Minimally-Processed Pineapple (Ananas comosus, var. ‘Queen Victoria’) during Cold Storage: Fungi in the Leading Role
Source: Microorganisms. 2020 Jan 28;8(2):185. doi: 10.3390/microorganisms8020185 (PMC7074791; doi:10.3390/microorganisms8020185)
Supplement: Supplementary file 1 [file microorganisms-08-00185-s001.pdf]

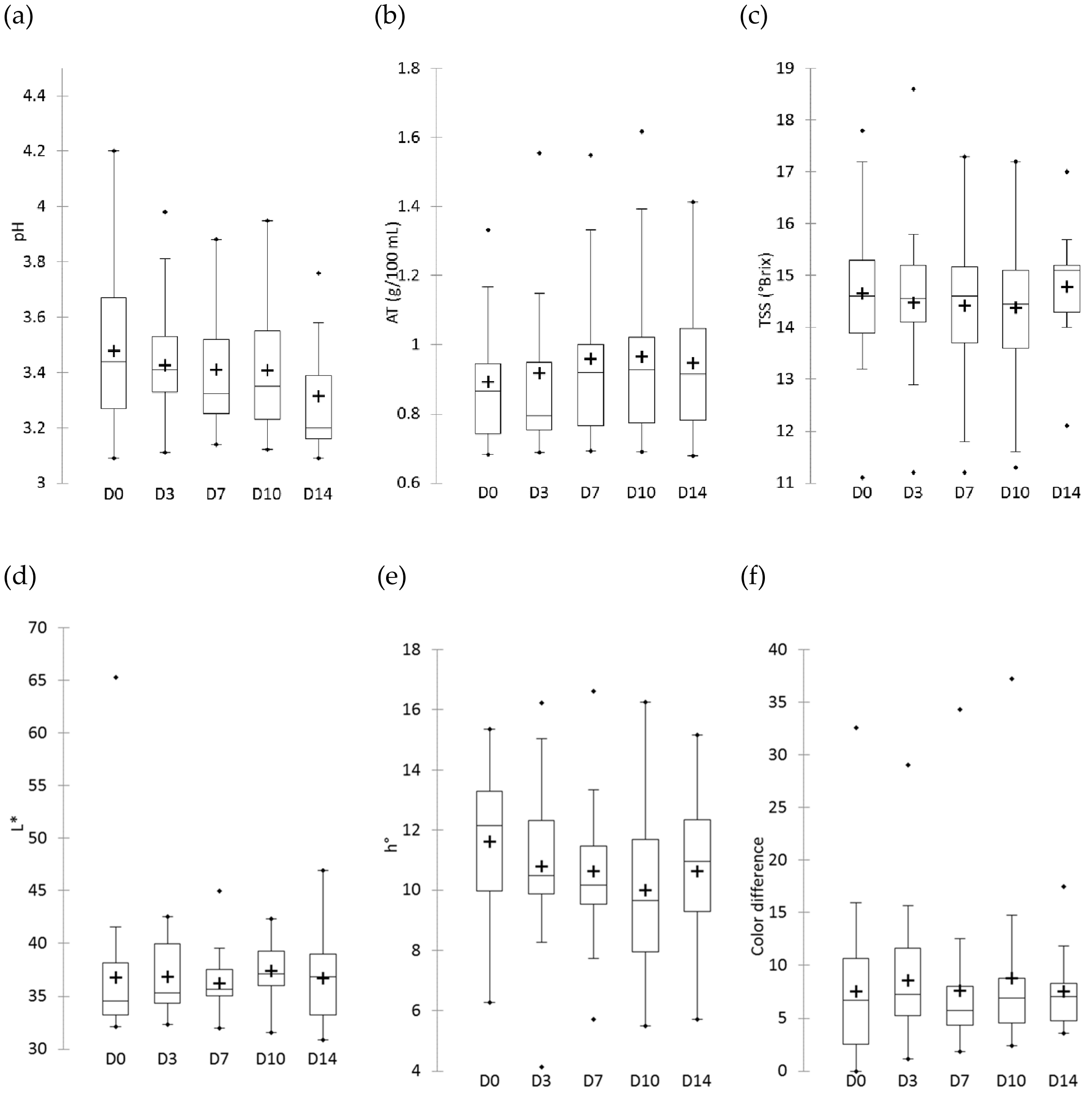

Figure S1. Values for **(a)** pH, **(b)** TA, **(c)** TSS, **(d)** L*, **(e)** h° and **(f)** ΔE of 25 minimally-processed pineapple batches during storage at 4°C. Time of storage varies from 0 (D0), 3 (D3), 7 (D7), 10, (D10) and 14 (D14) days. + indicates the mean value, and black dots the outliers
